# Hyperglycemia related to high-dose glucocorticoid use in noncritically ill patients

**DOI:** 10.1186/1758-5996-5-18

**Published:** 2013-04-04

**Authors:** Jose Gerardo Gonzalez-Gonzalez, Leonor Guadalupe Mireles-Zavala, Rene Rodriguez-Gutierrez, David Gomez-Almaguer, Fernando Javier Lavalle-Gonzalez, Hector Eloy Tamez-Perez, Gerardo Gonzalez-Saldivar, Jesus Zacarias Villarreal-Perez

**Affiliations:** 1 Endocrinology Sevice, “Dr. José E. González” University Hospital and School of Medicine, Universidad Autónoma de Nuevo León, Ave. Madero y Gonzalitos s/n, Colonia Mitras Centro, Monterrey, Nuevo Leon 64460, Mexico; 2 Department of Internal Medicine, “Dr. José E. González” University Hospital and School of Medicine, Universidad Autónoma de Nuevo León, Ave. Madero y Gonzalitos s/n, Colonia Mitras Centro, Monterrey, Nuevo Leon 64460, Mexico; 3 Hematology Service, “Dr. José E. González” University Hospital and School of Medicine, Universidad Autónoma de Nuevo León, Ave. Madero y Gonzalitos s/n, Colonia Mitras Centro, Monterrey, Nuevo Leon 64460, Mexico

**Keywords:** Diabetes, Secondary diabetes, Glucocorticoid-induced diabetes, Glucocorticoid, Hyperglycemia, Drug-induced diabetes

## Abstract

**Background:**

Glucocorticoids commonly cause drug-induced diabetes. This association is well recognized but available evidence does not answer clinically relevant issues in subjects without diabetes.

**Methods:**

Thirty-five individuals without diabetes with a recent diagnosis of acute lymphoblastic leukemia or non-Hodgkin’s lymphoma on high-dose glucocorticoid therapy were studied. Close systematic monitoring of fasting and postprandial glycemia and fasting insulin determinations, HOMA-insulin resistance and HOMA β-cell function were performed. The primary objective was to define the incidence of secondary diabetes in patients treated with high-dose glucocorticoids. Secondary objectives were to specify the intensity, the moment it appears and the evolution of hyperglycemia, in addition to the risk factors, mechanisms and impact of continuous and cyclical glucocorticoids on the development of hyperglycemia.

**Results:**

Mean age of patients was 38.4 ± 18.7 years. The incidence of diabetes was 40.6% and was found after the first week; half the time it occurred between the second and fourth. Two-thirds spontaneously normalized by eight weeks. Continuous glucocorticoid administration had a higher incidence of fasting hyperglycemia (*P* = 0.003). Mean peak insulin levels were significantly higher in cases of diabetes.

**Conclusions:**

High-dose prednisone for 2 to 3 months produced an elevated incidence of diabetes, usually with mild hyperglycemia occurring between the second and fourth week, normalizing spontaneously in all cases. Hyperglycemia was more frequent with continuous doses and occurred in cases with increased insulin resistance. The clinical and therapeutic characteristics of our participants, who were otherwise healthy, could represent the clinical setting of many patients with illness from other medical areas that might require high doses of GC for six to twelve weeks.

## Background

Drug-induced hyperglycemia is a clinical condition that can occur as a result of impaired insulin secretion or action or the destruction of pancreatic beta cells [1]. The administration of glucocorticoids (GC) is a common cause [2]. Gulliford et al. found 2% of newly diagnosed cases of diabetes mellitus (DM) due to orally administered GC [3]. Its use is associated with multiple side effects with DM being one of the most important [4,5]. In cases of DM prior to the use of GC, worsening of glycemic control is typical; however, in patients without DM, glucocorticoids may transiently or permanently induce hyperglycemia [5-7].

Two mechanisms are predominantly responsible for hyperglycemia secondary to GC: a decrease in insulin secretion and insulin sensitivity [5,7,8]. Although the link between GC and hyperglycemia was identified more than 50 years ago, clinically relevant questions remain unanswered [2,5,7,9]. The incidence of DM secondary to the use of GC, the profile of subjects in whom it occurs, the consequences of comorbidities, the moment it becomes apparent, its intensity, and its evolution in the short and medium term are not well defined. An incidence of 2% to 50% has been reported [3,10]. Published studies have used methodologies accountable for this broad range. Most are retrospective with a very large GC dose range, different DM diagnostic criteria, comorbid conditions that affect insulin sensitivity, and with no description of the course of hyperglycemia [3,5,9-16]. It is important to find answers to clinical scenarios in which GC are used. A common setting is the administration of high dose (>1 mg/kg/day) GC for one to three months in a patient without comorbidities and in whom DM can occur. Obtaining this information would allow appropriate diagnostic and therapeutic strategies [5,9,17].

With the aim of defining the incidence of secondary DM, we designed a prospective, longitudinal, observational study in patients with a recent diagnosis of hematological diseases without DM and in whom treatment with GC would be initiated. Secondary end points were to identify the intensity of hyperglycemia, the moment it develops, its progression, and the risk factors, possible mechanisms responsible for the occurrence of hyperglycemia, and the impact of a continuous glucocorticoid regimen (CGC) *vs.* a cyclic glucocorticoid regimen (CyGC).

## Subjects and methods

### Subjects

We studied 35 consecutive patients of the Hematology Service of the “Dr. José E. Gonzalez” University Hospital. Approval was obtained from the Institutional Review Board and all participants signed an informed consent. Male or female patients between 18 and 80 years of age with acute lymphoblastic leukemia (ALL) or non-Hodgkin’s lymphoma (NHL) who needed treatment with a dose of GC equal to or greater than 1 mg/kg/day of prednisone were included. Pregnant women, those with a personal history of DM and a fasting plasma glucose level ≥5.60 mmol/L at enrollment and use of GC for more than 7 days in the last two years or the use of medications that cause secondary DM were excluded.

### Study protocol

After baseline blood sampling, patients were started on GC treatment for their hematological disease. The possibilities were twofold: CGC or CyGC with prednisone. In CGC, the study period was eight weeks, taking a weekly blood sample for determination of fasting plasma glucose and serum insulin. In addition, participants kept a weekly record of three 2-hour postprandial capillary glucose determinations. For ALL cases, a prednisone dose of 100 mg/day was used daily only during the first 6 weeks (CGC). In NHL cases, follow-up was for 12 weeks with a fasting plasma glucose and serum insulin determination every 20 days, at baseline and then before each GC cycle (CyGC). In addition, participants kept a weekly record of one fasting and three 2-hour postprandial capillary glucose determinations. The cycle was 100 mg of prednisone daily for five days followed by 15 days off. This was repeated five times. To contrast this with the other group (CGC), the participants were also evaluated at eight weeks. At this moment, CGC subjects had received 42 days of GC and were evaluated 14 days after interruption. Those with CyGC had received three cycles, with 11 days free from GC.

### Diagnostic categories of hyperglycemia

Fasting hyperglycemia was defined as a plasma glucose ≥5.60 mmol/L. Prediabetes and DM were defined as a fasting plasma glucose ≥5.60 and <7.00 mmol/L and ≥7.00 mmol/L, respectively [1]. A postprandial capillary glycemia ≥11.10 mmol/L was considered abnormal [18]. Any fasting plasma glucose result ≥5.60 mmol/L was confirmed with a new determination. Any postprandial capillary glycemia ≥11.10 mmol/L was confirmed by another capillary determination. Any participant who developed prediabetes and DM during the study, was classified as having DM.

### Risk factors for diabetes mellitus

The risk factors considered were age ≥45 years, a body mass index (BMI) ≥25 kg/m^2^, a waist-hip ratio (WHR) ≥0.8, hypertension, acanthosis nigricans and a history of type 2 DM in a first-degree relative.

### Measurements

For the measurement of plasma glucose, the glucose oxidase method (Adiva 1650 Chemistry System, Bayer, Leverkueusen, Germany: Intraassay CV <2%) was used. For serum insulin an electrochemiluminescence immunoassay (Roche Diagnostics, Indianapolis, IN; Intraassay CV <2%) was used. Capillary glycemia was determined with test strips and a glucometer (Accu-Chek Active, Roche Diagnostics Corp., Basel, Switzerland). The homeostatic model assessment (HOMA) insulin resistance (IR) and HOMA β-cell function (β-cell) were calculated as previously described [19].

### Statistical analysis

All results are reported as means ± standard deviation unless otherwise indicated. We conducted a descriptive statistical analysis for quantitative variables, measures of central tendency and dispersion. In the case of quantitative variables, frequencies were obtained. In quantitative comparative data, we used Student’s *t* test, paired or unpaired, depending on the case. For dichotomous variables we used the *X*^2^ test. For the magnitude of association we used odds ratio. A *P ≤* 0.05 or an odds ratio different from 1 was considered significant. The statistical analysis was performed with IBM SPSS Statistics 17.0 (SPSS, Inc., Armonk, NY).

## Results

### Study population

Thirty-five patients were enrolled and 32 (91%) completed the study. Three participants were excluded, two due to death because of hematological complications and a third due to poor protocol compliance. Table 1 shows the baseline clinical characteristics of the total group and by CGC and CyGC groups. Mean age was 38.4 years. Fifty-three percent were women. NHL was the most common disease. Mean BMI was 27.1 ± 5.7 kg/m^2^ and 63% had a family history of type 2 DM. There were no statistical differences between groups (CGC *vs.* CyCG) at baseline.

**Table 1:** Baseline clinical and therapeutic characteristics of studied patients (n = 32)

	**Total (n = 32)**	**CGC**^**1 **^**(ALL) (n = 12)**	**CyGC**^**2 **^**(NHL) (n = 20)**	***P *^ 1 *vs. *2**
**Age** (yrs.)*	38.4 ± 18.7	34.3 ± 21.5	40.8 ± 16.7	0.35
**Gender,** n (%)				
Male	15 (46.8)	6 (50)	9 (45)	0.82
Female	17 (53.2)	6 (50)	11 (55)	0.76
**BMI**^†^				
<20	2 (6.3)	1 (8.3)	1 (5)	0.88
20-24.9	7 (21.9)	2 (16.7)	5 (25)	0.45
25 – 29.9	14 (43.7)	4 (33.3)	10 (50)	0.23
≥30	9 (28.1)	5 (41.7)	4 (20)	0.19
**W/H ratio,** n (%)				
≥0.8	29 (90.6)	9 (75)	20 (100)	0.81
<0.8	3 (9.4)	3 (25)	0	0.12
**PAD** mg/patient				
8 weeks	-	4,200	1,500	-
12 weeks	-	-	2,500	-
**AN,** n (%)	4 (12.5)	2 (16.7)	2 (10)	0.70
**Family history DM,** n (%)	20 (62.5)	10 (83.3)	10 (50)	0.11

*Values are mean ± SD.

**^** Significant *P* value = ≤ 0.05.

^†^BMI = weight in kilograms by the square of the height in meters.

CGC, Continuous glucocorticoid regimen; CyGC, cyclic glucocorticoid regimen; ALL, acute lymphoblastic leukemia; NHL, non-Hodgkin’s lymphoma; GC, Glucocorticoids; BMI, Body-Mass Index; W/H ratio, waist to hip ratio; PAD, prednisone accumulative dose; AN, acanthosis nigricans; and DM, diabetes mellitus.

### Development of hyperglycemia and secondary diabetes mellitus

a) Fasting glycemia

The incidence of fasting plasma hyperglycemia at 8 weeks was 68.7% (22 out of 32 cases) (range, 5.60 to 17.72 mmol/L) (Table 2). The incidence of DM and prediabetes by fasting glycemia criteria was 34.3% in each category (11 of 32). All cases without fasting hyperglycemia (10 cases) were on CyGC. The cumulative dose of prednisone was significantly higher in those who developed hyperglycemia (2972 ± 1376 *vs.* 1500 ± 0 mg, respectively) (*P* = 0.002). The cumulative dose of prednisone in DM cases was 3218 ± 1362 *vs.* 2727 ± 1410 and 1500 ± 0 mg/day in prediabetes and in the group without fasting hyperglycemia (*P* = 0.67 and 0.0008), respectively. Only one subject developed fasting hyperglycemia between 8 and 12 weeks with a glycemia of 6.66 mmol/L.

b) Postprandial capillary glycemia

By week eight, the incidence of postprandial capillary hyperglycemia was 15.6% (5 out of 32 cases) (Table 2). Three also had a fasting plasma glycemia ≥7.00 mmol/L. There were eight subjects with a fasting glycemia ≥7.00 mmol/L without postprandial hyperglycemia. At 12 weeks, no new cases of postprandial hyperglycemia occurred. The cumulative dose of prednisone for cases with and without postprandial hyperglycemia was not statistically different.

**Table 2:** Incidence and time of occurrence of the different categories of hyperglycemia at 8 weeks follow-up in all studied patients (n = 32)

**Category**	**Value**
**Non-hyperglycemic** (≤ 5.6 mmol/L)	
n (%)	10 (31.3)
Baseline fasting glycemia (mmol/L)	4.7 ± 0.6
Plasma glucose (mmol/L)	4.5 ± 0.8
Cyclic	10
Continuous	0
PAD (mg/patient)^1^	1500 ± 0
**Fasting hyperglycemia** (≥5.60 mmol/L)	
Incidence, n (%)	22 (68.7)
Baseline fasting glycemia (mmol/L)	4.8 ± 0.7
Plasma glucose (mmol/L)	7.08 ± 3.04
PAD (mg/patient)^2^	2972 ± 1376
Cyclic	10
Continuous	12
*P* value (1 *vs.* 2)	0.002*
**Prediabetes** (≥5.60- < 7.00 mmol/L)	
Incidence, n (%)	11 (34.3)
Baseline fasting glycemia (mmol/L)	4.6 ± 0.9
Plasma glucose (mmol/L)^3^	6.01 ± 0.35
PAD (mg/patient)^3^	2727 ± 1410
*P* value (1 *vs.* 3)	0.01*
**Prediabetes appearance** (week), n (%)	
First	6 (54.5)
Second to fourth	2 (18.1)
Fifth to eighth	3 (27.3)
**DM** (fasting glycemia ≥7.00 mmol/L)	
Incidence, n (%)	11 (34.3)
Baseline fasting glycemia (mmol/L)	5.1 ± 0.8
Plasma glucose (mmol/L)	8.90 ± 3.63
PAD (mg/patient) ^4^	3218 ± 1362
*P* value (1 *vs.* 4)	0.0008*
*P* value (3 *vs.* 4)	0.67
**DM occurrence** (week), n (%)	
First	0 (0)
Second to fourth	5 (45.5)
Fifth to eighth	6 (54.5)
**Postprandial hyperglycemia**	
Incidence, n^†^ (%)	5 (15.6)
Capillary Glycemia (mmol/L)	11.33 ± 0.30
PAD (mg/patient)	
Hyperglycemic cases^5^	2040 ± 1207
Non-hyperglycemic cases^6^	2600 ± 1351
*P* value (5 *vs.* 6)	0.39

All data are reported as total number of subjects and (%) or mean ± SD.

PAD, prednisone accumulative dose; DM, diabetes mellitus GC; Glucocorticoid.

*Significant *P* value ≤ 0.05.

^†^Three cases also had a fasting plasma glucose ≥7.00 mmol/L.

### Characterization of hyperglycemia

Prediabetes occurred in 34.3% (11 out of 32 cases) (Table 2). Fifty-four percent of the cases occurred in the first week. DM also occurred in 11 of 32 cases (34.3%). Five cases occurred between the second and fourth week (9.25 ± 2.90 mmol/L), and six afterwards (7.37 ± 0.40 mmol/L). Eleven cases had prediabetes without developing DM. Treatment was not started in any case and values normalized spontaneously.

Of the 22 participants with fasting hyperglycemia, 15 normalized by the eighth week. The other seven cases had a fasting glycemia between 5.60 and 7.94 mmol/L (6.50 ± 0.93 mmol/L). Two belonged to the CGC group (7.94 and 6.72 mmol/L). Of the other five cases, at week 12, three normalized and two continued with prediabetes. All cases of postprandial hyperglycemia normalized at 8 weeks.

### Risk factors

A multiple logistic regression analysis was performed, which detected that only the CGC scheme (OR 2.0, 95% CI, 1.29–3.1) was an independent risk factor for developing DM (*P* = 0.003). Other risk factors studied did not show significant differences (Table 3).

**Table 3:** Risk factors associated to glucocorticoid-induced hyperglycemia

	**OR**	**95% CI**	P*
**Age**	0.75	0.15 – 3.7	0.73
**BMI**^†^	0.33	0.66 – 1.47	0.13
**W/H Ratio**	0.89	0.134 – 5.88	0.90
**AN**	0.40	0.48 – 3.34	0.39
**DM FH**	2.14	0.464 – 9.90	0.33
**GC Regimen (CGC *vs. *CyGC)**	2.00	1.29 – 3.1	0.003*

*Significant *P* value ≤ 0.05.

^†^BMI = weight in kilograms by the square of the height in meters.

OR, Odds Ratio; CI 95%, 95% Confidence Interval; BMI, Body Mass Index; W/H, waist-to-hip ratio; AN, acanthosis nigricans; DM FM, diabetes mellitus family history; GC, glucocorticoid; CGC, continuous glucocorticoid regimen, and CyGC, Cyclic Glucocorticoid regimen.

### CGC vs. CyGC

The incidence of fasting hyperglycemia at eight weeks was 100% with CGC *vs.* 50% with CyGC (*P* = 0.003) (Table 4). Of these, seven and four had DM, respectively. Five of the seven cases of DM in CGC occurred between the second and fourth week. All four cases in the CyGC group occurred after the fourth week. There was no significant difference in the incidence of postprandial hyperglycemia between groups (*P* = 0.62).

**Table 4:** Incidence and categories of hyperglycemia in accordance to scheme of GC therapy

**Variable**	**CGC n = 12**	**CyGC n = 20**	P*
PAD (mg/patient)			
8 weeks	4200	1500	-
12 weeks	-	2500	-
**Fasting hyperglycemia** (≥5.60 mmol/L)			
Incidence, n (%)	12 (100)	10 (50)	0.003*
Plasma glucose (mmol/L)	8.22 ± 3.79	6.50 ± 0.64	0.16
**Prediabetes** (≥5.60- < 7.00 mmol/L)			
Incidence, n (%)	5 (41.6)	6 (30)	0.51
**DM** (fasting glycemia ≥7.00 mmol/L)			
Incidence, n (%)	7 (58.3)	4 (20)	0.22
Plasma glucose (mmol/L)	9.83 ± 4.39	7.11 ± 0.15	0.26
**DM occurrence** - week**,** n (%)			
First	0 (0)	0 (0)	-
Second to fourth	5 (71.4)	0 (0)	-
Fifth to eighth	2 (28.5)	4 (100)	-
**Postprandial hyperglycemia**			
Incidence, n (%)	1 (8.3)	4 (20)	0.62

All data are reported in total number of subjects and (%) or mean ± SD.

CGC, continuous glucocorticoid regimen; CyGC, cyclic glucocorticoid regimen; PAD, prednisone accumulative dose; DM, diabetes mellitus.

*Significant *P* value ≤0.05.

Basal insulin, HOMA-IR and ß-cell were not statistically different between groups (Table 5). The mean values of serum insulin levels increased during follow-up. When the two groups were compared, there was no significant difference. Average baseline insulin, HOMA-IR, and HOMA ß-cell of the participants with and without DM in the CGC group compared with the values of the CyGC group were not statistically different. However, the average peak value for insulin in both groups (CGC and CyGC) was statistically significant when cases with and without DM were compared (*P* = 0.02 and 0.01, respectively). Average peak values of insulin between CGC and CyGC groups showed significant differences (238.21 ± 73.62 *vs.* 150.71 ± 70.14 pmol/L, respectively; *P* = 0.002).

**Table 5:** Insulin, HOMA-IR and HOMA ß-cell values at baseline and during follow up by GC scheme*

**Variable**	**CGC n =12**	**CyGC n = 20**	***P *^**
**Baseline**			
Insulin (pmol/L)	64.59 ± 17.36	53.48 ± 18.75	0.12
HOMA-IR	2.2 ± 0.79	1.73 ± 0.79	0.11
HOMA (β-cell)	112.8 ± 47.8	113.3 ± 63.2	0.98
**Insulin, follow-up week** (pmol/L)			
1	105.56 ± 66.67	-	-
2	125.70 ± 75.01	-	-
3	142.37 ± 57.64	106.95 ± 59.73	0.11
4	154.87 ± 88.20	-	-
5	156.96 ± 86.81	-	-
6	169.46 ± 109.73	125.70 ± 73.62	0.19
8	129.87 ± 68.75	-	-
9	-	120.15 ± 70.84	-
12	-	96.53 ± 40.28	-
**Insulin** (pmol/L) [n]			
With DM^1^	63.20 ± 15.97	61.46 ± 20.14	0.88
Without DM^2^	66.67 ± 21.32	51.39 ± 18.75	0.15
*P* value (1 *vs.* 2)	0.75	0.39	-
**HOMA-IR**			
With DM^3^	2.20 ± 0.74	2.14 ± 0.96	0.91
Without DM^4^	2.21 ± 0.94	1.62 ± 0.75	0.17
*P* value (3 *vs.4*)	0.99	0.35	-
**HOMA- β-cell**			
With DM^5^	98.8 ± 21.9	135.3 ± 102	0.36
Without DM^6^	132 ± 68.9	107 ± 53.1	0.40
p value (5 *vs.6*)	0.24	0.45	-
**MMII**^†^ pmol/L(%)			
With DM [week]^7^	145.84(231%) [6]	154.18(251%) [9]	0.76
Without DM [week]^8^	82.64 (116%) [4]	56.25 (111%) [6]	0.37
p value (7 *vs.* 8)	0.02*	0.01*	-

All data reported as total number of subjects and (%) or mean ± SD.

CGC, continuous glucocorticoid regimen; CyGC, cyclic glucocorticoid regimen; DM, diabetes mellitus; HOMA-IR, Homeostasis Model Assessment Insulin Resistance; HOMA ß-cell, Homeostasis Model Assessment Beta Cell Function; MMII, mean maximum insulin increase.

*Significant *P* value ≤ 0.05.

*p*^ continuous *vs.* cyclic.

HOMA-IR = [Fasting Insulin (μU/ml) x Fasting Glucose (mmol/L)]/22.5.

HOMA- ß-cell = [20 x Fasting Insulin (μU/ml)]/[Fasting Glucose (mmol/L)]-3.5.

^†^Mean absolute maximum value.

## Discussion

In a noncritically ill adult population, using a high dose of prednisone (>1 mg/kg/day) for 6 to 12 weeks, in two classical schemes of GC administration, under close monitoring of fasting and postprandial glycemia, we identified a high incidence of prediabetes and DM. At eight weeks, by fasting and postprandial glycemia, a DM incidence of 40.6% was found. For prediabetes, the incidence was 34.3%. The incidence of DM did not increase at 12 weeks. Unlike other series, in this study fasting glycemia was more sensitive in identifying DM than postprandial determinations [5,11,12,20]. Only two cases that had DM by postprandial hyperglycemia did not meet fasting plasma glucose DM criteria. Series of patients on high doses of GC have found a DM incidence in the range of our study, between 12.6% and 52% [11,13-16]. Despite methodological problems such as: different populations with heterogeneous comorbidities, diverse diagnostic methods for DM, a lack of close monitoring of glycemia and varying patterns of high doses of GC, the incidence of DM in most of these studies was between 15% and 25%, less than the incidence of DM of 40.6% that we found in our study [11,13-16]. This difference can be attributed mainly to the retrospective design and lack of close systematic monitoring of fasting and postprandial glycemia. Contrary to our study, the study with the highest incidence (52%) is a retrospective series in which all cases of DM were detected solely by postprandial glycemia [11].

When starting high-dose GC in a subject without DM, there are two interesting clinical issues for making appropriate decisions: the intensity that hyperglycemia can reach and when it may occur. With regard to the intensity, acute complications of DM have been reported in a few critically ill cases [14,21-24]. In this study, most of the time hyperglycemia was not intense, deciding, under close supervision, not to start management of hyperglycemia, which allowed recording its evolution. The average fasting glycemia of patients with DM was 8.89 mmol/L and only four of 32 patients had a fasting glycemia greater than 8.33 mmol/L. Besides our study, no other has prospectively and closely evaluated the intensity of hyperglycemia. The second issue is the time it takes for hyperglycemia to occur. Of the 13 cases of DM, none occurred in the first or after the eighth week, and half appeared between the second and fourth week. Therefore, it is advisable to search for it during the second, fourth and sixth week of exposure to high doses of GC. This finding is consistent with the results of other authors that have also identified most cases of DM in early stages of exposure to GC [12,14,15]. Recently, a clinical practice guideline on the management of hyperglycemia in hospitalized patients, suggested that monitoring of glycemia in individuals without diabetes exposed to GC can be interrupted if glycemia is less than 7.78 mmol/L for a period of at least 24 to 48 hours [25]. Our results in noncritically ill patients show that hyperglycemia occurs after the first week or even until the eighth week of exposure to GC, coinciding with findings in other retrospective series [12,14,15]. It is likely that other comorbidities that are present in hospitalized patients alter the action or secretion of insulin and anticipate the onset of hyperglycemia.

Another question that has not been carefully evaluated in previous studies is the course of hyperglycemia. In this study, two-thirds of subjects with prediabetes or DM normalized spontaneously by the eighth week. The rest, at 12 weeks, showed normal glycemia or were in the range of prediabetes. This spontaneous remission in the majority of the participants could be attributed to the absence of other comorbidities, which might impair insulin secretion or action. Many cases of severe hyperglycemia related to GC use, occurred in subjects with an aggregated acute disease [14,23,24].

Some authors have proposed that in individuals with a prediabetes state, GC could precipitate the onset of DM [2,5,6]. In our study, mean basal serum insulin and HOMA-IR were not different between participants with CGC or CyGC or when comparing subjects with and without DM. These findings suggest that at the beginning there was no difference in insulin resistance between the groups. However, in participants on CGC and CyGC who developed DM there was a significant increase in insulin secretion compared with those who did not. This finding suggests that the primary mechanism involved in the onset of DM is most likely, increased insulin resistance, associated to a limited compensatory ability of the pancreas.

## Conclusion

In a noncritically ill population, without other comorbidities, the use of high doses of GC for two to three months, as a CGC or CyGC dose, caused a high incidence of DM with non-severe hyperglycemia, most often with CGC, which appeared after the second week of GC treatment and resolved spontaneously without treatment in the next six weeks. Even though these results reflect glucose behavior in two specific hematological disorders, the clinical and therapeutic characteristics of our participants, who were otherwise healthy, could represent the clinical setting of many patients of other medical areas that might require high doses of GC for six to twelve weeks. It would be useful to perform prospective studies in other clinical situations that use GC, such as patients with multiple comorbidities, long-term low dose GC, or in critically ill patients.

## Abbreviations

DM: Diabetes mellitus; GC: Glucocorticoids; ALL: Acute lymphoblastic leukemia; NHL: Non-Hodgkin’s lymphoma; CGC: Continuous glucocorticoid regimen; CyGC: Cyclic glucocorticoid regimen; BMI: Body mass index; HOMA IR: Homeostatic model assessment insulin resistance; HOMA β-cell: Homeostatic model assessment β-cell function; WHR: Waist-to-hip ratio.

## Competing interests

The authors declare they have no competing interests.

## Authors’ contributions

JGGG, LGMZ, RRG served as the principal investigators and contributed to study design, data collection, and manuscript preparation. DGA and FJLG contributed to study design, data collection and manuscript preparation. HETP participated in statistical analysis and manuscript preparation. GSG contributed to data collection and manuscript preparation. JZVP contributed to the coordination and helped draft the manuscript. All authors read and approved the final manuscript.
